# 

**DOI:** 10.1192/bjb.2024.77

**Published:** 2025-04

**Authors:** Ellen Fallows

**Affiliations:** British Society of Lifestyle Medicine, Haddington, UK. Email: ellen.fallows@bslm.org.uk

**Figure:**
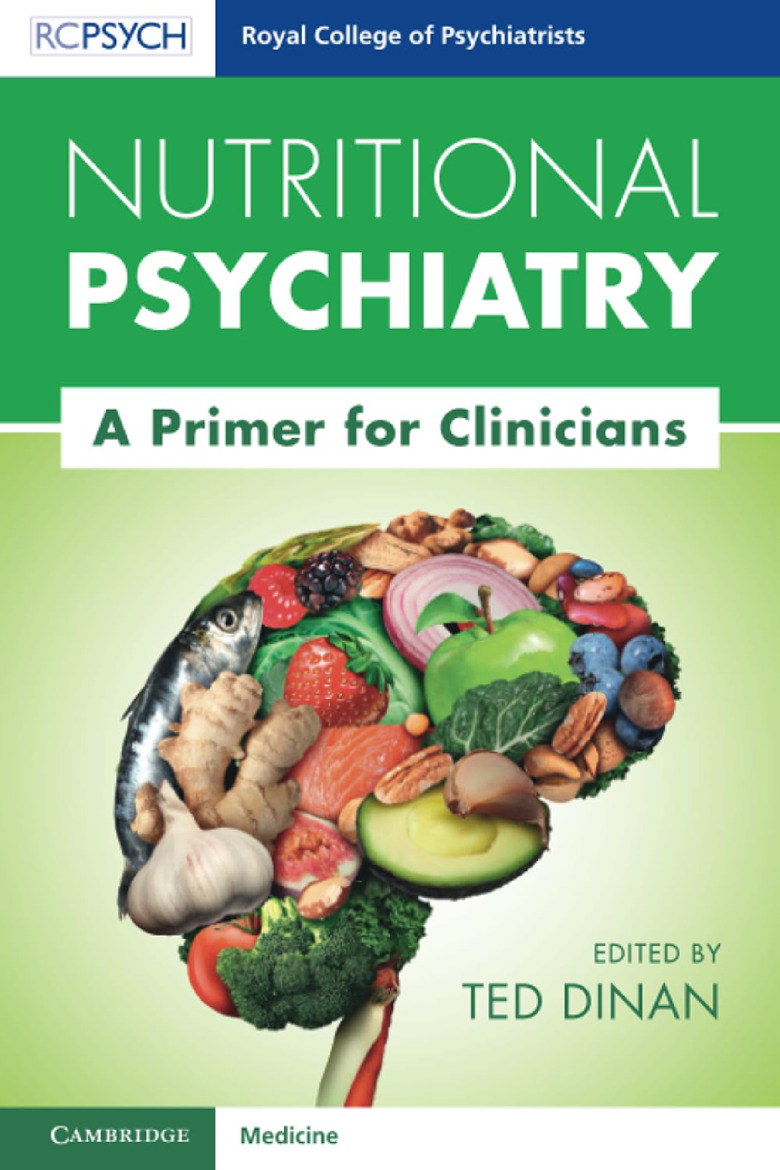

*Nutritional Psychiatry* marks a paradigm change in the field of psychiatry as one of the first textbooks in the emerging discipline of ‘Lifestyle Psychiatry’. This accessible guide will be of huge interest to a wide range of clinicians, including psychiatrists, GPs, health coaches, IAPT/mental health workers and researchers. Professor Ted Dinan and his authors explain the latest research and its practical application. The book's core messages are that we should move away from a reductionist focus on food as calories, macro and micronutrients, to a more complex view on the overall quality of diet. Secondly, that food has been neglected as a therapeutic tool in Psychiatry, despite its huge potential, because it is ‘one of the major modulators of the gut microbiome and bioactive substances produced by [it] have significant impact on brain function and mental health’ and that, ‘whilst our own inherited genome is stable throughout our lifetime, our microbial genes are large, diverse, adaptable and infinitely modifiable’, challenging our similarly reductionist view of genes as destiny.

*Nutritional Psychiatry* covers the basic principles of nutrition and the role of the gut–microbiota–brain axis in mental health, in addition to how dietary patterns, including the Mediterranean diet and fermented food, affect brain health. The book ends on the needs of specific groups, including children, adolescents and the elderly.

Inflammation is a common theme throughout the book, which explains that those with mental illness have chronic low-grade inflammation correlating with the likelihood of treatment response and severity of symptoms. The authors cover some surprising facts; that those with depression have an increased abundance of inflammatory bacterial taxa whose number correlate with symptom severity; that those with schizophrenia have gut microbiome disturbances, which when transferred through stools using ‘faecal microbial transfer – FMT’ into mice with no microbiome, result in changes in mouse brain neurochemistry and behaviour.

Psychobiotic treatments, including prebiotic, postbiotic, parabiotic (dead bacteria), symbiotic, FMT and diet interventions are reviewed. Frustratingly, the authors explain that most interventional trials have been small, short, yet to be reproduced and more often in rodent models.

For some clinical readers, a more stepwise introduction of complex concepts around the gut microbiome would have been useful, in addition to covering the harmful effects of modern dietary components such as ultra-processed foods and contaminants such as emulsifiers, artificial sweeteners, food contaminants (heavy metals/PFAS, etc.) and antibiotics.

With rising rates of mental illness and food insecurity, clinicians and policy-makers urgently need accessible but evidence-based manuals on how best to teach about and use food to improve mental health. *Nutritional Psychiatry* is a well-written and hugely welcome start in a neglected arena.

